# Organic–Inorganic Hybrid Materials

**DOI:** 10.3390/polym13010086

**Published:** 2020-12-28

**Authors:** Jesús-María García-Martínez, Emilia P. Collar

**Affiliations:** Polymer Engineering Group (GIP), Polymer Science and Technology Institute (ICTP), Spanish Council for Scientific Research (CSIC), C/Juan de la Cierva, 3, 28006 Madrid, Spain

According to the IUPAC (International Union of Pure and Applied Chemistry), a hybrid material is that composed of an intimate mixture of inorganic components, organic components, or both types of components which usually interpenetrate on scales of less than 1 μm [1]. This definition matches the subject of all the articles published in this special issue. Thus, the concept of organic–inorganic hybrid materials can be applied to a broad number of approaches. In the same way, the so-called organic–inorganic materials can be revealed as multi-component compounds having at least one of their organic (the polymer) or inorganic component in the sub-micrometric and, more usually, in the nano-metric size domain [2]. Further, the organic–inorganic hybrid systems can be classified into two classes named class I and Class II, depending on if the interaction of the phases is weak or strong, respectively [2]. The latter depends on the type of interactions: Van der Waals, hydrogen, electrostatic, and other interactions for Class I, and due to real chemical bonds between phases (Class II), the coexistence of both types of interactions (I and II) in the same system is possible [2]. In such a scenario, this issue includes a wide range of topics covering these definitions. Consequently, the organic–inorganic materials are revealed as multi-component compounds having at least one of their organic (the polymer) or inorganic component in the nano-metric size domain, and usually offers much better performance than their non-hybrid counterparts [2,3,4,5,6].

Under the above-mentioned premises, this special issue compiles 11 articles written by experts in the field and are devoted to the very different approaches coexisting in the field.

In this context, the first article by Echeverría, Rubio, and López [7] studies of thermo-reversible organic–inorganic hybrid gels by means of thermal and viscolestic properties. For such purposes, they used synthetized hybrid thermo-reversible gels by combining a metallo-organic polymer and isotactic polystyrene with the purpose of solving the poor mechanical properties of these types of compounds. They were able to determine three transitions upon heating (monotectic transition of the iPS gel, melting of the iPS gel, and melting of the metal-organic polymer gel) suggesting that the two polymers are formed independently in the hybrid gel, concluding that these hybrid gels are interpenetrated organic/metallo-organic networks.

Li et al. [8] investigated the influence of incorporating polydimethylsiloxane on the properties of PA66 fiber fabrics, obtained by melt blend spinning, by producing fibers with excellent mechanical properties and reduced hot shrinkage, without remarkable effect on crystallization and melting behavior of PA66. Thus, the effect of the additive on the surface tension suggests the antifouling, waterproof, and stain-resistant ability of the fiber and its fabric. These results are compared with silicon-coated fibers, resulting in the proposition by the authors of the compounds having many properties besides being ecofriendly.

Related to the hot topic of fire resistance materials, Sheng, Lu, and co-workers report a study about it [9]. So, this article is devoted to fire and smoke suppressions of polyvinyl alcohol (PVA) aerogels in order to avoid the fire hazard that they present. For this purpose, the authors used a 2D transition-metal carbide for enhancing the flame-retardant performance of PVA/ammonium polyphosphate-based composites. The results demonstrated that the presence of the carbide in low amounts can boost the flame retardance of the composites even as to pass as V-0 rated. Furthermore, the authors demonstrate that the presence of the carbide greatly reduces the release of heat and smoke, proving the synergistic effect of the ammonium phosphate and the carbide used for the objective of the study.

An investigation about the changes observed on the ultimate properties of isopropylene–mesoporous silica nanocomposites obtained by extrusion caused by the effect of the polymers confinement within the mesostructures is the subject of the article by Cerrada and working group [10]. The study concludes the key role of pore size in the confinement of iPP chains within the nanometric mesostructures of the silicas used, affecting the thermal stability and the ultimate properties of the materials. The main conclusions of the work were the related effect of the silica on the polypropylene observed through the degradation and the rheological behavior of the composites, which is explained by variations in the inclusion of the polymer chains within the mesostructures of the silicas used. The latter was demonstrated by synchrotron SAXS measurements.

Since the carbonation of concrete is a prime problem in civil engineering, the paper by Sousa, Ferreira, and co-workers [11] is related to the reduction of this concrete degradation by the effect of organic–inorganic hybrid films for the potential functionalization of optical fiber sensors to be applied in concrete structures. Virgin films and those doped with phenolphthalein were characterized by a series of techniques and the results obtained were highly promising regarding the properties under high alkaline scenarios causing concrete carbonation.

By considering the increasing importance of additive processing, the work by Peponi et al. [12] is focused on the fabrication of organic and inorganic poly(e-caprolactone)-based electrospun fibers in order to obtain different nanocomposite mats based on poly(e-caprolactone), PCL, reinforced with a series of both organic and inorganic nanoparticles (just 1%). As organic particles, the authors used cellulose nanocrystals (produced by them) and commercial chitosan and graphene, while the inorganic ones were silver and hydroxyapatite particles. The authors found no appreciable differences in terms of thermal parameters. The authors conclude that while the flexibility of the mats increases for whatever type of particle incorporated, the stiffness of the system increases only in the case of silver or graphene particle is present in the mat.

The effect of the dispersing agents with an ionic or steric action on the interactions between hollow glass microspheres and an epoxy-polyamide resin on composite materials for nautical applications are studied by Dodero, Vicini, and colleagues [13]. The composites are evaluated by means of the rheology of the un-crosslinked material as well as mechanical and morphological properties of the crosslinked composites. The authors conclude that steric dispersing agents offer much better compatibility than ionic ones based on their hindrance capability, and so a better-performing composite with a less-marked Payne effect is obtained. The authors claim to be the pioneers in studying the role of dispersing agents for this type of material in comparison to the merely empirical approaches followed for the preparation of fillers for this type of composite.

Smitthipong and coworkers [14] studied the performance of nano- and micro-calcium carbonate fillers in the final properties of un-crosslinked natural rubber compounds, by paying attention the high cost reduction for industry and the half reinforcing character of this type of fillers promoting better mechanical properties. The authors found a similar tendency of results with the independence of the nano or micro character of the nano-filler used; they also show the importance and influence of the specific surface area. However, the authors conclude the effect of the specific surface area on the final properties, as well as the theoretical models which agree with the results. Additionally, they showed (through time–temperature superposition master curves) where the higher filled composites exhibited lower free volume. Finally, they conclude about the real possibility of using nano-calcium carbonate material for the production of tailor rubber-based products.

Since bio-based nanocomposites have emerged as a hot topic, Garrigos, Puglia, and colleagues [15] have studied the effect of chlorophyll hybrid nanopigments obtained by combining chlorophyll dye (obtained from agro-food wastes), calcined hydrotalcite, and montmorillonite nanoclay, on the final color and thermomechanical properties, by using statistical design of experiments, DOEs. In this way, the authors were capable of identifying the more important parameters influencing the mechanical, thermal, structural, morphological, and color properties of the bionanocomposites studied. Hence, the potential use of green natural dyes from broccoli wastes, adsorbed into nanoclays, have been proven to be very interesting in the development of naturally colored bionanocomposites.

Attending to the fact that the interphase between the organic and the inorganic components of the hybrid material is what governs their ultimate behavior, García-Martínez and Collar [16] investigated the combined effect of the reinforcement and the interfacial agent in a polypropylene-based composite. In the article, the authors studied mica platelets and a polymer waste interfacial modifier consisting of a grafted atactic polypropylene with attached p-phenylen-bis-maleamic acid polar groups, by paying attention to the changes of the glass transition temperature. By using Box–Wilson experimental designs, the article focuses on the prediction of how this parameter changes as a function of both the interfacial agent and the nano-reinforcement by taking into consideration the complex character of the iPP/aPP-*p*PBMA/mica system, wherein the interaction between the components will define the final behavior, and that the glass transition is a design threshold for the ultimate properties of parts based in this type of organic–inorganic hybrid materials. Hence, the final purpose of the work is the prediction and interpretation of the effect of both variables on this key parameter, being identified the same critical points as by other properties such as flexural, tensile, impact, and thermal properties, as determined previously by the authors [16].

Finally, by focusing the attention on the fact that is the adjustment of the organic–inorganic interfacial chemical environment which plays a key role for the separation performance of composite materials, Miau, Wu et al. [17] focused their attention on the regulation about how a polyvinyl alcohol/sulfonated nano-TiO_2_ hybrid membranes interface promotes diffusion dialysis. For such purposes, the authors investigated the behavior of a series of hybrid membranes fabricated by combining polyvinyl alcohol (PVA) and sulfonated nano-TiO_2_ (SNT). In fact, they regulate the effects of the interfacial chemical surroundings on ion transfer as a function of the SNT content to affect the dialysis coefficients of both the hydroxyl ions and the separation factors in order to be of an adequate interval as to make the sulfonic groups in the interfacial regions and the hydroxyl groups present in the PVA main chain. This can have important roles during the transport of sodium and hydroxyl ions. Moreover, the authors identified a membrane with optimal performance in the case of 3% of SNT.

Heterogeneous materials based on organic polymers (as a whole or as a part) can play an essential roles in the fight against COVID-19. In fact, they can be key materials for fabricating preventive and isolation clothing for the medical teams in close contact with ill patients, for life support equipment, containment, and transport thereof. Additionally, organic–inorganic hybrid materials remain the realm for other diverse applications such as controlled dosing of drugs, membrane systems, multi-component packaging, and a myriad of accessories and sanitary items in general. The challenge of continually attending to new demands remains. The latter constitutes the prime motivation and concern for hybrid materials research. This extensively applies to the works compiled in this special issue.

We thank all the authors for their contributions and encourage them to pursue with their research.
